# The Extracellular Matrix and Vesicles Modulate the Breast Tumor Microenvironment

**DOI:** 10.3390/bioengineering7040124

**Published:** 2020-10-11

**Authors:** Jun Yang, Gokhan Bahcecioglu, Pinar Zorlutuna

**Affiliations:** 1 Department of Chemical and Biomolecular Engineering, University of Notre Dame, Notre Dame, IN 46556, USA; jyang26@nd.edu; 2 Department of Aerospace and Mechanical Engineering, University of Notre Dame, Notre Dame, IN 46556, USA; gbahceci@nd.edu; 3 Bioengineering Graduate Program, University of Notre Dame, Notre Dame, IN 46556, USA; 4 Harper Cancer Research Institute, University of Notre Dame, Notre Dame, IN 46556, USA

**Keywords:** breast cancer, extracellular matrix, extracellular vesicles

## Abstract

Emerging evidence has shown multiple roles of the tumor microenvironment (TME) components, specifically the extracellular matrix (ECM), in breast cancer development, progression, and metastasis. Aside from the biophysical properties and biochemical composition of the breast ECM, the signaling molecules are extremely important in maintaining homeostasis, and in the breast TME, they serve as the key components that facilitate tumor progression and immune evasion. Extracellular vesicles (EVs), the mediators that convey messages between the cells and their microenvironment through signaling molecules, have just started to capture attention in breast cancer research. In this comprehensive review, we first provide an overview of the impact of ECM in breast cancer progression as well as the alterations occurring in the TME during this process. The critical importance of EVs and their biomolecular contents in breast cancer progression and metastasis are also discussed. Finally, we discuss the potential biomedical or clinical applications of these extracellular components, as well as how they impact treatment outcomes.

## 1. Introduction

Breast cancer is the most common type of malignancy in women. According to the American Cancer Society, 276,480 new female cases are estimated to emerge in the United States, with over 40,000 deaths in 2020 [1]. Although significant advances have been made in breast cancer diagnosis and treatment, including surgical removal of the primary tumor, metastasis of breast tumors to distal organs such as bone, lung and liver [2] remains the main cause of cancer-related deaths [3]. While the effect of genetic and structural dysregulations in cells on cancer progression and invasion has widely been documented [4,5], the role of the tumor microenvironment (TME) has just started to gain interest [6,7].

Emerging evidence indicates that the extracellular matrix (ECM), a complex, evolving network of proteins and signaling molecules, plays a role in breast cancer progression and metastasis [8,9]. While the tumor leads to substantial changes in the structure and composition of the ECM, the ECM significantly contributes to breast cancer progression as well. These changes can potentially lead to metastatic spread or influence therapeutic outcomes [10]. While the effect of ECM structure, biochemical composition, and biomechanical properties on breast cancer development and progression has widely been studied, the role of extracellular vesicles (EVs) has just started to capture attention in the cancer field [11].

EVs are small, membrane-derived particles excreted by cells to the extracellular space. They are integral components of ECM [11] and serve as mediators in multiple biochemical signaling cascades. They transport biomolecules between cells and tissues and as such could play a vital role in breast cancer progression and metastasis [12]. More recently, growing insights into EVs have further enhanced our understanding of the breast TME. However, previous review papers have focused on the biology of EVs and their role in intercellular communications, and few have covered their role in breast cancer development [10,11].

In this review, we give an overview of the role of ECM in breast TME modulation as well as breast cancer progression, and then describe the specific importance of EVs and their contents in breast cancer progression and metastasis. Finally, we discuss the therapeutic potential of the ECM and EV components in breast cancer. This review provides an introduction to how the ECM and EVs modulate the breast TME in breast cancer, and how their components can be used in translational applications. 

## 2. Extracellular Matrix in Breast Cancer

**Figure 1:**
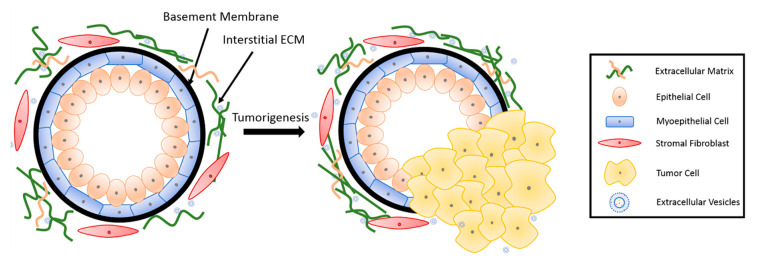
Breast tissue undergoing tumorigenesis. The basement membrane is a thin layer of pericellular matrix separating the epithelium and the stroma. Following tumorigenesis, a microenvironment is created, supporting tumor progression. Tumor cells surpass the basement membrane, which becomes more permissive in the tumor microenvironment (TME), invade the stroma, and eventually metastasize to distant sites through vasculature.

The basic mammary structure consists of luminal epithelial cells lining a central lumen, surrounded by a layer of myoepithelial cells, the stroma, and the basement membrane that separates the epithelium from the stroma (Figure 1). The basement membrane and the stroma make up an important part of the ECM in the mammary gland. The basement membrane is a thin layer of matrix mainly composed of type IV collagen, laminin and entactin [13]. Stromal ECM, which mainly contains type I collagen, fibronectin, laminins, and glycoproteins, serves as a structural scaffold that maintains breast tissue integrity and sustainability [14]. However, the role of ECM is way more significant than simply providing structural support. It plays multiple roles in regulating cell behavior in the breast tissue, such as survival, proliferation, differentiation [15], invasion [16,17], as well as immune responses [18]. Furthermore, the ECM mediates stromal–epithelial communication and serves as a guide that regulates breast development [10]. 

In recent decades, a growing body of research has revealed an important role of the ECM in breast cancer progression and metastasis [8,9]. During tumorigenesis, the structure and composition of the ECM is also significantly altered, further contributing to cancer progression [10]. In this section, we will discuss the influence of both the biophysical properties and biochemical composition of ECM on breast cancer progression and metastasis, as well as drug resistance. Moreover, we will discuss alterations taking place in the ECM during breast cancer development.

### 2.1. Role of Mechanical and Biophysical Properties of ECM in Breast Cancer

The ECM provides mechanical support to cells and guides them through mechanical stimuli. The cells adjust their behavior and remodel their microenvironment as a result of these forces [19]. These mechanical cues, including ECM density and stiffness, alter mechanotransduction signaling [20,21] and thus the protein [22] and miRNA [23] expression in cells. These alterations influence cell behavior including cell morphogenesis [24], stem cell differentiation [25,26,27], and cancer-associated fibroblast (CAF) activation [11,28], thereby potentiating and stimulating aggressive behaviors in malignant epithelial cells. For example, in a meta-analysis study, the risk of breast cancer demonstrated a compelling increase of up to 4–5-fold in women with 75% mammographic density compared to those with less than 10% density [29].

Stiffness is a well-known regulator of breast cancer cell behavior. ECM stiffness leads to profound changes in cancer cell growth, metastatic potential, as well as chemotherapeutic responses [30]. Collagen crosslinking also increases the ECM stiffness, and thus promotes tumor metastasis [31]. The collagen fibril bending stiffness of 3D collagen matrices was demonstrated to direct the spreading and clustering of breast cancer cells [32]. A previous study by Yue et al. [21] revealed that the influence of breast cancer cells on stromal cells was stiffness-dependent; breast cancer cells reduced the degree of adipogenesis only on stiff substrates. A more recent study showed that scaffold stiffness exerts its impact on breast tumor cell invasion through EGFR-linked Mena upregulation and matrix remodeling [33], altering matrix organization. Additionally, tissue stiffness regulates integrin-linked kinase (ILK) expression to control stem-like breast cancer cells under hypoxic conditions [7]. Tissue mechanical properties also modulate miR-18a expression to reduce PTEN and HOXA9 levels, and subsequently regulate cancer invasive progression [23]. In the same vein, stromal miR-200s-regulated ECM stiffness contributes to breast cancer metastasis through CAF activation [28]. 

The organization of the ECM is another factor influencing breast cancer cell behavior. Interestingly, collagen alignment was reported to promote migration in invasive breast cancer cells more than in non-invasive cells [34,35]. The stromal tissue is rich in type I collagen, and the collagen network in the stroma serves as a physical barrier against cancer cell invasion [36]. On the other hand, normal epithelial cells grown on type I collagen bind to type I collagen and go through epithelial-to-mesenchymal transition (EMT) [37]. Type I collagen leads to increased secretion of matrix metalloproteinases (MMPs) which facilitate ECM degradation, and induces invasive behavior [38]. Upon ECM fragmentation by MMPs, ECM bound growth factors are released [39] and a path is opened for the cancer cells to migrate through. 

### 2.2. Role of Biochemical Composition of ECM in Breast Cancer

Aside from the mechanical and biophysical properties, the biochemical composition of the ECM also has a significant impact on breast tumor progression, metastasis, and response to treatments. Growing evidence indicates that many ECM proteins serve a major functional role in breast cancer progression, metastatic niche construction, and metastatic growth promotion. A study from Staren group [40] proved that ECM proteins, such as vitronectin and fibronectin, can enhance the metastatic potential of breast cancer cells by regulating cell adhesion and migration with integrin subunits. Many ECM proteins, including collagen, osteonectin, and hyaluronic acid, are involved in breast cancer development. Type I collagen poses a versatile role in breast cancer development. Fibronectin expression level in breast cancer cells is significantly associated with a higher probability of metastasis [41]. Upregulation of fibronectin also promotes formation of the pre-metastatic niche [8]. Proteoglycans various pathological processes, including cancer progression and metastasis [42]. Proteoglycan expression is altered in the breast TME during tumor development, and such an alteration affects cancer cell growth, adhesion, signaling, migration and angiogenesis [43]. A higher expression of proteoglycan in breast cancer cells is often correlated with increased tumor risk [44], grade [45] and size [44], directing the cells toward metastasis [46].

Cytokines and growth factors are becoming a significant part of breast-cancer-related studies. Many cytokines are considered as prognostic markers in breast cancer. These cytokines also impact breast cancer progression. Table 1 summarizes the principal cytokines involved in the prevention or progression of breast cancer. Transforming growth factor β (TGF-β), one of the most significant and widely studied cytokines in cancer research, is pro-tumorigenic and involved in breast cancer cell proliferation [47]. Tumor necrosis factor α (TNF-α) enhances the dendritic cell (DC) antitumor effect, inhibits growth and promotes apoptosis of breast cancer cells [48]. Fibroblast growth factor acidic (acidic FGF) is involved in the estrogen-independent and antiestrogen-resistant growth of MCF7 breast cancer cells [40]. Many interleukins (IL) are involved in the cellular immunity and communication of stromal cells with breast cancer cells [49]. IL-1α is known to promote metastasis [50], as it contributes to the induction of pro-metastatic genes in breast cancer [51]. IL-6 induces T cell and B cell differentiation, stimulates cytotoxic T cells and assists in killer cell activation to promote antitumor activity [52], demonstrating its therapeutic potential.

Under normal conditions, breast tissue maintains homeostasis. As the ECM starts to change and becomes suitable for cancer development, disruption of homeostasis follows. While tumor cells create their own microenvironment by remodeling the ECM, the TME also impacts cancer cell behavior, leading to a more aggressive phenotype. In a pathological microenvironment, collagen fibers tend to become relatively straight, forming a more organized alignment [35]. ECM protein components could be degraded or modified by cancer-associated enzymes. MMPs are an important category of enzymes involved in ECM degradation and remodeling, and play a role in tumor cell invasiveness. MMP1, 2, 7–11, 13, 14, and 16 are constitutively expressed in tumor cell lines but not in normal breast epithelial cells [33]. MMP expression alters the rigidity, porosity, and many other characteristics of the ECM, facilitating cell migration and invasion. Breast cancer cells can activate the surrounding stromal cells to create CAFs or cancer-associated adipocytes (CAAs), which remodel the ECM and promote tumor invasiveness [77]. Breast cancer cells also modify the dynamics of stromal fibronectin and collagen interactions, with the help of MMPs [33]. The sequestered pro-angiogenic factors are released as the ECM remodels, further facilitating downstream breast cancer invasion.

Nucleic acid cargo is another influential component of ECM. Lots of effort has been put into identifying the nucleic acid profiles of the breast TME. MicroRNAs (miRNAs) are a family of small-size, non-coding RNA molecules that function as post-transcriptional gene regulators, playing roles in cancer proliferation and invasion [78]. In breast tissue, miRNAs regulate the expression of cytokines and growth factors [79] that can affect ECM composition and pave the way for pathogenesis. miRNAs are often dysregulated in breast cancer [80]. Researchers found out that a single-nucleotide polymorphism (SNP) with miR-196a2 is associated with a decreased risk of cancer [81], while an SNP in miR-146a has been reported to be linked to earlier onset of breast cancer [82]. In a study with more than 1000 patients, the upregulation of miR-103/107 was shown to be associated with metastasis and poor outcome of breast cancer patients [83]. The downregulation of miR-210 was reported to be inversely correlated with cancer aggressiveness and metastatic capability [84]. In a study by Song group [85] with 32 patients, miR-21 was shown to target MMP3 expression to regulate breast cancer invasion. Stromal miR-200s might also regulate CAF activation and ECM remodeling to promote breast cancer cell invasion [21].

### 2.3. The ECM as a Physical Barrier for Breast Cancer Treatment 

The biophysical and biochemical properties of the breast significantly impact the treatment outcome of the patient. ECM components affect the penetration of immune cells, antibodies and drugs into tumor sites [86]. The dense and stiff collagen network may also serve as a physical barrier against drug penetration [87]. Hence, collagenase treatment can significantly enhance drug penetration for collagen-rich tumors [88]. Glycoseaminoglycans, such as hyaluronic acid and chondroitin sulfate, may also limit drug penetration to the tumor site.

In the meanwhile, interactions between cancer cells and the ECM can drastically affect the sensitivity of cells to apoptosis and their response to chemotherapeutic drugs. ECM proteins mediate drug resistance in breast cancer in multiple therapies. Stromal-derived MMPs are involved in tamoxifen resistance. Loss of function experiments showed that MMPs facilitated the release of heparin-bound EGF, which further regulated cell behavior, resulting in the paracrine induction of 4-OH-tamoxifen resistance through EGFR and PI3K/AKT pathways [89,90]. In HER2-positive breast cancer, ECM/integrin signaling promoted drug resistance to combination therapy aiming at HER2 and PI3K inhibition [91]. Doxorubicin was shown to be more effective against MDA-MB-231 cells when ECM-cell signaling was disrupted by inhibiting β1-integrin [92]. 

miRNAs are also involved in the modulation of chemotherapy responses. The dysregulation of miRNAs also affects the success of therapeutic interventions. miR-19, miR-21 and miR-203 expression in the breast results in resistance to chemotherapy [80]. Moreover, the expression of miR-34 and miR-155 suppresses radiotherapy sensitivity [80]. miRNA-34a has been reported to be associated with docetaxel resistance in human breast cancer cells [93].

## 3. Extracellular Vesicles in the Breast TME

**Figure 2:**
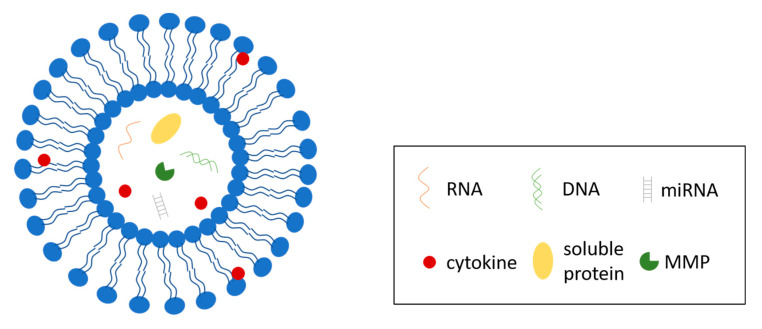
Structure of extracellular vesicles (EVs). EVs are lipid-bilayer enclosed vesicles secreted by cells to regulate multiple cellular processes. EVs contain and transport a variety of biomolecules, including RNAs, DNAs, miRNAs, soluble proteins, and a system of cytokines.

Extracellular vesicles are small, lipid-bilayer membrane-derived particles released from a cell into the extracellular space (Figure 2). EVs were first reported by Erwin Chargaff and Randolph West in 1946 [94], and the nature of these particles was further enunciated by Peter Wolf [95]. In recent years, EVs have gained increasing interest because of their connection to multiple types of cells, tissues and pathological conditions, such as cancer, as well as their ability to transport proteins, nucleic acids, lipids and other molecules that are important in many signaling events. EVs are present in body fluids [96,97] and serve as transport vehicles and protective envelopes for their cargos in the extracellular environment [98], delivering messages between cells and their surroundings. According to recent studies, EVs can be considered as integral and functional components of the ECM [11]. In breast tissue, EVs facilitate the transportation of bioactive molecules that could play significant roles in tumor progression and invasion [12]. In this section, we will discuss breast-cancer-associated EVs, their cargos, and some of their effects on breast cancer progression.

### 3.1. Extracellular Vesicles

The EVs involved in breast cancer development include various vesicle subtypes. Table 2 summarizes the basic similarities and differences between the various types of EVs. The most commonly studied category of EVs, exosomes, are shed from most normal or diseased cells [99] and originate from multivesicular bodies in a cell that fuses with the plasma membrane. Although often considered together with exosomes, microvesicles are another vesicle population with distinct morphologies and sizes and are shed through different exocytosis mechanisms [100]. While the sizes of exosomes generally range from 50 to 80 nm, microvesicles can go beyond 1 μm in diameter [101]. Other extracellular vesicle subtypes include the apoptotic bodies, which are shed from cells undergoing apoptosis, and the large oncosomes, which are produced by cancer cells and promote cancer progression [102]. ECM-bound vesicles are another subtype of EVs, which recently have been shown to play a potential role in cancer progression [103]. 

EVs serve as messengers between the tumor and its microenvironment, facilitating the transportation of bioactive molecules that further facilitate tumor progression and metastases. Therefore, the isolation and characterization of cancer-derived vesicles is of the upmost importance to solve their roles in breast cancer. A lot of studies in the cancer research field focus on the isolation and characterization of new EV subtypes. Recent studies have identified a subset of ECM-bound vesicles [103] that mediate physiologic and pathologic processes in multiple diseases, including cancer. Their research into matrix-bound vesicles demonstrated that these vesicles and their miRNA cargo mediated the effects of the ECM bioscaffold on macrophage phenotype and impacted macrophage function [104].

### 3.2. Extracellular Vesicle Cargos in Breast Cancer and Their Effects

EVs carry cargos that mediate communication between cells and their microenvironment. In clinical applications, EVs are also proposed to be used as biomarker reservoirs in liquid biopsy [105]. They carry biomolecules, including proteins, nucleic acids, lipids, and other bioactive molecules from cells to the ECM. Communication between cancer cells and normal stromal cells is important for the development of cancer [106]. EVs have shown to be involved in tumorigenesis and the non-cell regulation of cancer cells [106]. Increasing evidence has indicated that EVs derived from cancer cells provide a means of delivering cellular messages to other cells within the tumor, as well as the primary TME and any metastatic niches. MCF10A cells are able to uptake EVs secreted by MDA-MB-231 cells, which would induce the regulation of E-cadherin and secretion of MMPs, and promote invasion of these non-tumorigenic epithelial cells [107]. Proteomic analysis of the MDA-MB-231 breast adenocarcinoma cell derived exosome-like vesicles identified 179 proteins and 32 protein isoforms, in correspondence to 22 genes, that were more abundant in EVs compared to whole-cell lysate [108]. These vesicles deliver multifunctional proteins, including those regulating cell proliferation, cell cycle, and cell death (14-3-3 epsilon and PDC6IP), to adjacent neoplastic or non-tumoral cells to promote or activate malignancy. EVs derived from breast cancer cells and associated TGF-β and VEGF secretion drive myofibroblastic differentiation of adipose-derived stem cells (ASCs), activate MAPK signaling pathways in ASCs and promote ASC pro-angiogenic behavior [109]. Feng et al. [110] identified a unique 90 kDa form of VEGF delivered through MDA-MB-231 cell-secreted microvesicles that activates VEGF receptors and induces angiogenesis in tumors. Campos et al. [111] reported that caveolin-1 containing EVs promoted breast cancer cell malignancy by adhesion protein transportation. A recent study also unveiled a system of cytokines encapsulated in EVs that are capable of eliciting their biological effects [112], which could be an interesting aspect in future research on EVs in the breast TME.

In addition, EVs contain small noncoding RNAs, including microRNAs (miRNAs), which can contribute to the tumorigenesis and malignancy of cancer cells [106]. miR-105 released from breast-cancer-associated EVs suppresses ZO-1 expression in endothelial cells, weakening cell–cell adhesion and promoting metastasis [113]. Breast cancer cells secrete EVs containing miR-122, which serves to reprogram glucose metabolism in premetastatic niche and promote metastasis [114]. Furthermore, metastatic breast cancer cells that colonized in the brain release EVs containing miR-181c, which facilitates blood–brain barrier destruction and mediates brain metastasis [115]. Research indicates that miR-134 in EVs can inhibit triple-negative breast cancer invasion and boost anti-Hsp90 drug sensitivity [116]. All miR-200 family members, including miR-200a, miR-200b, miR-200c, miR-429 and miR-141 are found to be enriched in EVs secreted by breast cancer cells. EVs containing miR-200 promote breast cancer cell metastasis and deliver metastatic message to distal tumor cells [117].

Recent studies have revealed the impact of cancer-cell-derived EVs on drug resistance. Triple-negative breast cancer (TNBC)-derived EVs significantly alter the genetic profiling of MCF10A cells and promote cell proliferation and resistance to Docetaxel and Doxorubicin treatments [118]. HER2 is overexpressed in around 25% of breast cancers [119], making HER2-targeting drugs a potential anti-cancer treatment for many cancer patients. A recent study indicates that EVs from neuromedin U (NmU)-overexpressing cells contain higher levels of TGFβ1 and PD-L1 and promote immune evasion [120]. Promoted immune evasion is associated with resistance to HER-2 targeted anti-tumor therapies.

## 4. Therapeutics Potential of Major ECM and EV Components in Breast Cancer

Thriving insights into the biological and pathologic roles of ECM and EVs in breast cancer indicated the potential of ECM and EV components and downstream signaling pathways in clinical applications. ECM components that promote tumor progression and metastasis can be appealing targets for breast cancer therapies, while increments of tumor suppressing components can enhance the anti-tumor effect of the microenvironment and potentially facilitate therapeutic outputs. β-aminopropionitrile (BAPN), the inhibitor of the ECM crosslinking enzyme lysyl oxidase, promotes tumor latency and inhibits tumor growth [121]. Treatment with DX-2400, an MMP14-blocking antibody, reduces hypoxia and SMAD2/3 signaling to inhibit tumor growth in murine models and synergistically promotes the effect of radiotherapy [122]. IL-2 administration enhances the antitumor effect of DCs, resulting in complete tumor eradication and extended cancer-free survival [123]. More recently, Bei group have constructed chimeric antigen receptor CAR-147 and proved that CAR-147 macrophages target breast-tumor ECM to inhibit tumor growth in vivo [124]. Aside from being widely considered as biomarker reservoirs in liquid biopsy for clinical cancer diagnosis, EVs have drawn considerable interests for their ability to carry functional biomolecules and their potential in drug delivery [125,126] and regenerative medicine [127]. In a recent study [128], EVs were modified with aptamers to improve the performance of siRNA delivery to treat breast cancer. Kuroda et al. [129] produced engineered EVs with a superior delivery efficiency of anti-tumor miRNA to breast cancer cells.

## 5. Concluding Remarks

The ECM of breast cancer cells is a dynamic network tuned by multiple factors, such as aging, obesity, and many pathologic conditions, including cancer. Major alterations occur in the ECM as breast cancer progresses as well. Changes in biochemical profiles and biomechanical properties regulate ECM functions and modulate breast tumor progression and metastasis. ECM stiffness increases the tendency of tumorigenesis and cancer progression. ECM components, such as proteins and nucleic acids, regulate cancer cell behavior through multiple pathways. As a functional part of the ECM, EVs carry bioactive molecules and deliver messages between cells and their surrounding environment. Breast cancer cells rely on certain ECM components, including EV-derived biomolecules, to survive, invade, or turn into dormancy or go through apoptosis. In clinical settings, many ECM or EV components can be used as prognostic biomarkers to facilitate early stage diagnosis and therapeutic arrangement. Moreover, with increasing knowledge of ECM composition and functions, ECM components appear to be appealing drug targets. Blockade of ECM-mediated signals that promote tumor progression and metastasis demonstrated promising results in multiple in vivo studies. The critical role of ECM and EVs in breast cancer development calls for more in-depth studies into understanding their functions and their relationship with the primary tumor. Enhanced understanding of their interactions may further promote translation into clinical applications.

## Figures and Tables

**Table 1:** Common cytokines involved in breast cancer.

Cytokine Type	Role in Breast Cancer	Ref.
IL-1 family	Promote angiogenesis, tumor proliferation and local tumor invasion	[53,54]
IL-4	Inhibit breast cancer cell growth	[55,56]
IL-6	Promote tumor cell proliferation, induce T- and B-cell activation	[57]
IL-8	Promote tumor growth and metastasis	[58,59]
IL-10	Inhibit tumor growth, induce drug resistance	[60,61,62]
IL-12	Inhibit breast cancer cell proliferation and invasion	[34,63]
IL-18	Inhibit metastasis	[64]
IL-33	Promote breast cancer cell proliferation	[65]
Type I Interferon (α,β)	Inhibit tumor proliferation and invasion	[66]
Interferon γ p	Promote breast cancer proliferation and invasion	[67]
TGF-β	Promote breast cancer cell proliferation	[47]
gp130	Promote breast cancer cell proliferation and invasion	[68]
TNF-α	Promote breast cancer metastasis	[69]
Vascular endothelial growth factor (VEGF)	Promote breast cancer metastasis	[70]
MMP-2	Stimulate breast cancer metastasis	[71]
Acidic FGF	Inhibit breast cancer proliferation	[40]
Platelet-derived growth factor (PDGF)-BB	Promote breast cancer cell invasion	[72]
Leukemia inhibitory factor (LIF)	Promote breast cancer cell proliferation and invasion	[73]
Cystatin C	Inhibit breast cancer cell proliferation	[74]
Resistin	Facilitate breast cancer progression and promote breast cancer metastasis	[75,76]

**Table 2:** Common Types of Extracellular Vesicles.

Vesicle Type	Size [102]	Common Functions
Exosome	50–100 nm	Modify the extracellular microenvironment Facilitate intercellular communication Regulate pathogenesis
Microvesicle	>200 nm	Modify the extracellular microenvironment Facilitate intercellular communication Regulate pathogenesis
Apoptotic body	>500 nm	Breakdown of apoptotic cells May facilitate intercellular signal transduction
Large oncosome	1–10 μm	Facilitate communication from cancer cells Modify the extracellular microenvironment Regulate cancer progression
